# Supplementary material for: Using scalable computer vision to automate high-throughput semiconductor characterization
Source: Nat Commun. 2024 Jun 11;15:4654. doi: 10.1038/s41467-024-48768-2 (PMC11166656; doi:10.1038/s41467-024-48768-2)
Supplement: Supplementary file 1 — Supplementary Information [file 41467_2024_48768_MOESM1_ESM.pdf]

Supplementary Information:
Using Scalable Computer Vision to Automate High-throughput Semiconductor Characterization

Alexander E. Siemenn[1*†], **Eunice Aissi**[1*†], Fang Sheng[1], Armi Tiihonen[1,2], Hamide Kavak[1,3], Basita Das[1], Tonio Buonassisi[1]

[1]Department of Mechanical Engineering, Massachusetts Institute of Technology, Cambridge, MA 02139, USA

[2]Department of Applied Physics, Aalto University, Espoo, 02150, Finland

[3]Department of Physics, Cukurova University, Adana, 01330, Turkiye

*Corresponding authors: {asiemenn@mit.edu, eunicea@mit.edu}

†Contributed equally.

Experiments

Processing Times

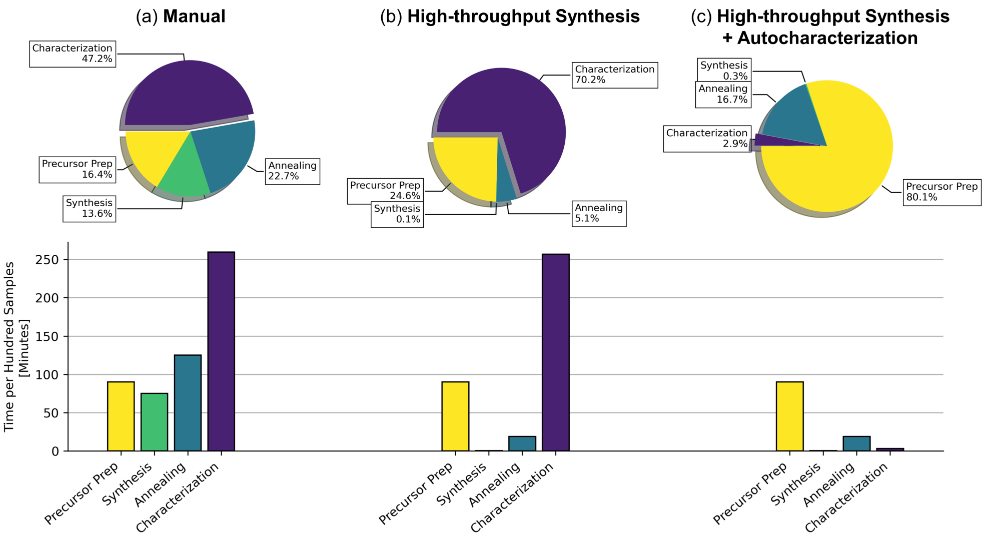

Fig. S-1: Minimum time to process 100 perovskite samples and compute band gap. The processing times are shown for three different scenarios: (a) manual synthesis manual and characterization, (b) high-throughput synthesis and manual characterization, and (c) high-throughput synthesis and high-throughput autocharacterization.

In this paper, we aim to achieve higher rate-matching between the synthesis and characterization of materials for high-throughput screening. In Figure S-1, the minimum processing time required to go from precursor to data is illustrated for perovskite samples. The minimum processing times to collect band gap data for 100

perovskite samples are shown and represent the times experimentally recorded during this study. Three different scenarios are represented that use combinations of both manual and high-throughput methods. The processing time to collect these band gap data is further broken down into four steps: (1) precursor preparation, (2) synthesis, (3) annealing, and (4) characterization. These are the minimum processing times as they do not account for sample transfer times or reloading, *e.g.*, moving a sample to the hotplate. In Figure S-1b, moving from manual to high-throughput synthesis results in a higher discrepancy of throughputs between synthesis and characterization, which bottlenecks the materials screening loop. However, in Figure S-1c, by using high-throughput autocharacterization, the rate of high-throughput synthesis is closely matched to that of high-throughput synthesis, in turn, enabling more efficient materials screening. The bottleneck then becomes precursor preparation, which is out of the scope of this study.

To detail the individual processing time contributions in this study, precursor preparation is always conducted manually and takes 90 minutes to make 100 samples worth of solution for all three scenarios. Synthesis takes 45 seconds per sample for manual spin coating and takes 20 seconds per 100 samples for high-throughput manufacturing. Annealing takes 10 minutes for manual thin films where 8 samples can fit on a single hot plate at a time. It takes 15 minutes for the thicker, high-throughput samples but all 100 samples can fit on a single hot plate. Characterization for computing band gap takes 255 minutes per 100 samples manually by a domain expert. It takes 3 minutes per 100 samples using the band gap extractor autocharacterization algorithm developed in this paper.

Experimental Reproducibility

Variability exists across samples manufactured using our high-throughput combinatorial printer, as the setup is designed for low-cost, scalable, and high-throughput screening rather than high-fidelity experiments. Although the purpose of the paper is not to highlight the perovskite manufacturing method but to focus on the rate matching of characterization, it is important to understand the sources of variability from experiment to experiment, as these sources of variability will arise for those who replicate the proposed approaches. Figure S-2 shows the post-degradation structural morphology differences between two of the same sample compositions across two separate batches, manufactured using the same printing conditions. Both batches were degraded under the same environmental conditions for 2 hours at 35°C, 40% relative humidity, and 0.5 suns of AM1.5 illumination (without UV). During crystallization, batch A achieved more uniform and compact grain boundaries, whereas the crystallization in sample B produced jagged boundaries, inducing more pathways for degradation. This crystallization mismatch explains the accelerated degradation noted in batch B relative to batch A. Samples that are prone to phase changes (*e.g.*, compositions near phase boundaries such as $0.0 \leq x \leq 0.15$, for $FA_{1-x}MA_xPbI_3$), may experience high sample-to-sample variances using any sample preparation approach. Therefore, the effects of these variations must be carefully considered in high-throughput manufacturing scenarios, where not every sample can be fully characterized at high fidelity. These morphological variations in the high-throughput manufacturing process must be further studied to ascertain better control over experimental reproducibility. In this

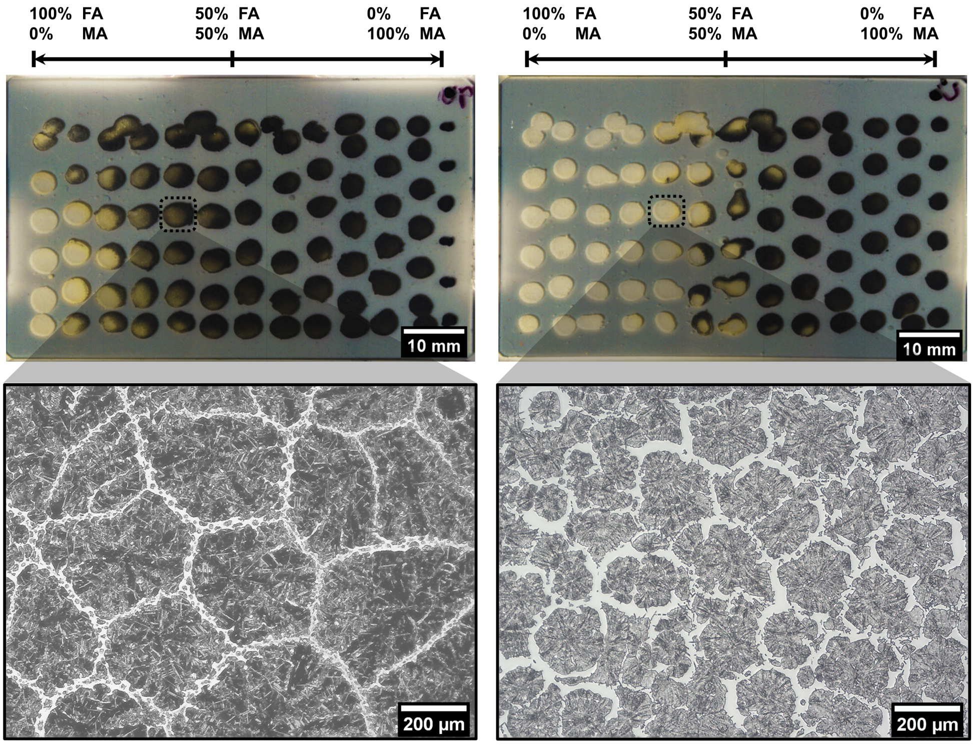

(a) Batch A

(b) Batch B

Fig. S-2: Optical microscopy of deposit morphology. Panels (a) and (b) illustrate the same compound of 67% formamidinium 33% methylammonium lead iodide ($FA_{0.67}MA_{0.33}PbI_3$) between two different batches after controlled degradation.

study, three batches of samples were selected with similar morphology in an attempt to minimize this variation and maintain the emphasis of the paper on the assessment of the proposed automatic characterization methods, rather than on the method of synthesis.

Material Characterization

Computer Vision Segmentation and Composition Mapping

Figures S-4a-b illustrate the process of going from a raw hyperspectral datacube ($\mathbf{\Omega}$) to computer vision-segmented data ($\mathbf{\Phi}$), which is used as input to the autocharacterization methods developed in this paper. An image, $\mathbf{\Omega}$, which can be in either Hyperspectral or RGB format, is segmented using Algorithm 1, producing the segmented pixels, $(\widehat{\mathbf{X}}, \widehat{\mathbf{Y}})$, and their corresponding set of reflectance values, $\mathbf{R}(\lambda)$. The matched sets of $(\widehat{\mathbf{X}}, \widehat{\mathbf{Y}})$ and $\mathbf{R}(\lambda)$ are denoted as $\mathbf{\Phi}$. The compositions of each deposited sample are then able to be mapped onto the segmented $\mathbf{\Phi}$ using the G-code raster

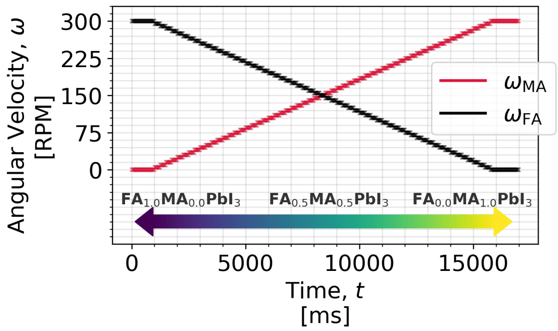

Fig. S-3: Perovskite synthesis pump velocity traces. Traces of motor velocity, ω, over time for each perovskite precursor, methylammonium lead iodide ($MAPbI_3$) and formamidinium lead iodide ($FAPbI_3$), for high-throughput combinatorial deposition as droplets.

path of the printer head, the pump speed traces from Figure S-3, and Equation 1. Figure S-4c illustrates this complete mapping of all material deposits with their derived compositions within the $FA_{1-x}MA_xPbI_3$ series.

Band Gap

The materials within the $FA_{1-x}MA_xPbI_3$ compositional series are direct band gap semiconductors [1, 2]. To compute the band gap of these materials, first, the reflectance spectra of all samples are measured using a hyperspectral camera (Resonon Pika L) that measures reflectance within the wavelength range $\lambda \in [380\text{ nm}, 1020\text{ nm}]$ at 2 nm resolution. Figure S-5 illustrates the measured reflectance spectra for all $N = 201$ samples in the paper, gathered using the computer vision-segmentation of the raw hyperspectral datacube, as shown in Figure S-4. Then, the reflectance spectra are converted to their corresponding absorption spectra using the Kubelka-Munk equation [3, 4] for sufficiently thick samples (the thickness of our samples is approximately 300 μm – which is considered sufficiently thick for reflectance measurement):

$$F(\mathbf{R}) = \frac{(1-\mathbf{R})^2}{2\mathbf{R}}, \tag{S-2}$$

where $\mathbf{R}$ is the reflectance for the entire range of λ for a given segmented pixel in the reflectance hypercube, $(\widehat{\mathbf{X}}, \widehat{\mathbf{Y}})$. Next, the Tauc curves are computed from $F(\mathbf{R})$ [5]:

$$(F(\mathbf{R}) \cdot \mathrm{h}\nu)^{1/\gamma} = \mathrm{B}(\mathrm{h}\nu - E_g), \tag{S-3}$$

where hν is energy ($\mathrm{h}\nu = \frac{1240}{\lambda}$), $\gamma = \frac{1}{2}$ for direct band gap and $\gamma = 2$ for indirect band gap, B is a constant that allows the band gap, E_g, to be the x-intercept of a regression fit line to the slope of Tauc curve. Hence, the following equation arises that enables computation of the direct band gap from the initial reflectance spectra by equating Equation S-4 to Equation S-3:

$$(F(\mathbf{R}) \cdot \mathrm{h}\nu)^2 = \left(\frac{(1-\mathbf{R})^2}{2\mathbf{R}} \cdot \frac{1240}{\lambda}\right)^2 = \left(\frac{620(1-\mathbf{R})^2}{\lambda\mathbf{R}}\right)^2. \tag{S-4}$$

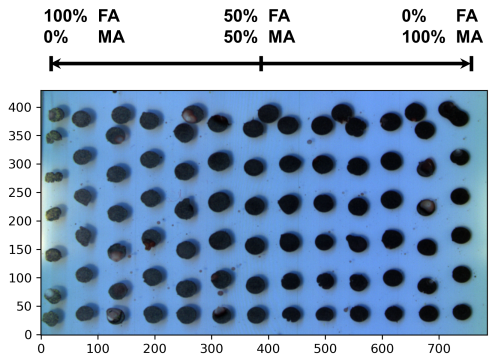

(a) Raw Image

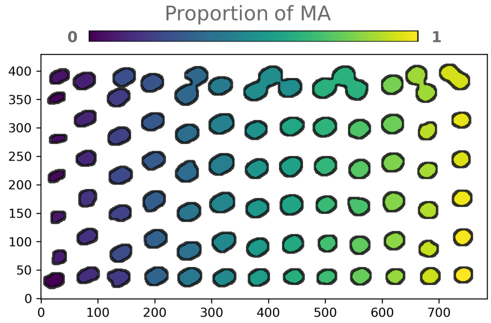

(b) Computer Vision-segmented Image

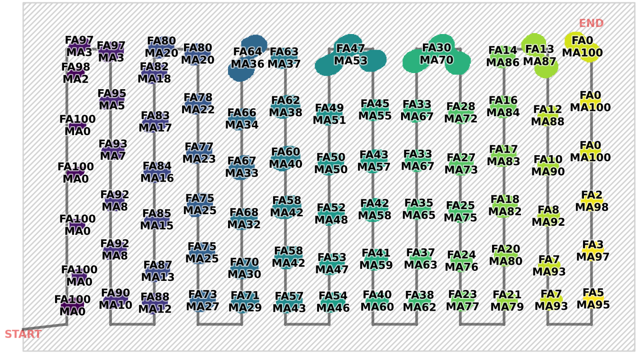

(c) Automated Composition Extraction Overlay

Fig. S-4: Computer vision segmentation and composition mapping procedure. (a) Raw image of the printed methylammonium (MA) and formamidinium (FA) mixed-cation perovskite system $FA_{1-x}MA_xPbI_3$. (b) Naive computer vision segmentation of the printed materials. (c) Segmented materials mapped to their corresponding proportion of MA and FA using extracted print head positions and motor velocity traces.

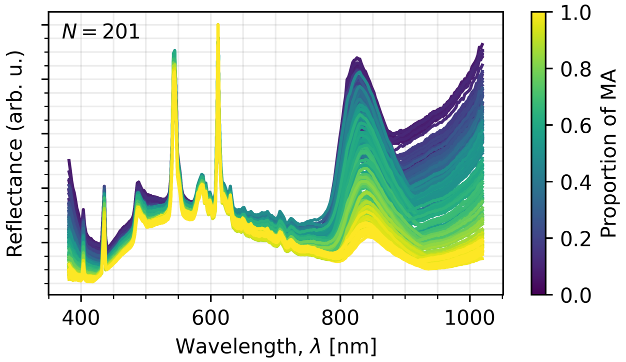

Fig. S-5: Hyperspectral reflectance of all $N = 201$ samples synthesized in this study, color mapped as a function of the proportion of methylammonium (MA) in the formamidinium (FA) and MA mixed-cation perovskite system $FA_{1-x}MA_xPbI_3$.

In this paper, we use the theory formulated above from optics to automatically compute the median band gap across all vision-segmented pixels $(\widehat{\mathbf{X}}, \widehat{\mathbf{Y}})$ for a given perovskite sample, as described by the autocharacterization algorithm illustrated in Figure 4. Currently, the autocharacterization algorithm is configured to operate only on materials with a single direct band gap. The band gaps computed using the automatic Tauc segmentation and regression RMSE minimization processes employed by the autocharacterization algorithms are benchmarked against the band gaps calculated manually by a domain expert. This band gap comparison between algorithm and expert is used to determine an accuracy metric for the algorithm, assuming the expert-calculated output as ground truth. The accuracy is calculated by taking the average of a binary 0/1 for all $N = 201$ samples, determined based on whether or not the differences between the automatic and the expert band gap values fall within a specified energy difference threshold (shown along the x-axis of Figure S-7b).

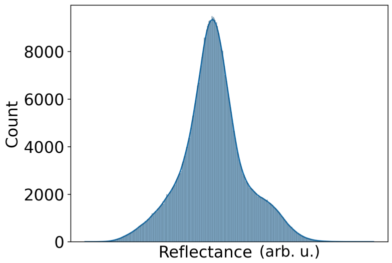

Fig. S-6: Distribution of reflectance values at the band gap for regression fitting. Histogram is shown for reflectances of all materials in this study.

Figure S-6 illustrates the distribution of reflectance values at the band gap slope for all material across all pixels in this study. At these slopes, the regression lines are

fit to compute the band gap for each material, as described in the Methods section. These data are shown to be normally distributed with no fat tails.

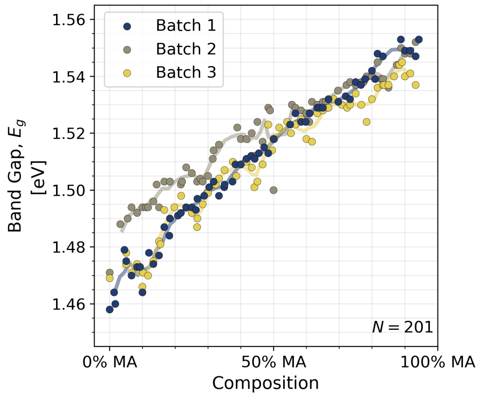

(a) Algorithm Batched Output

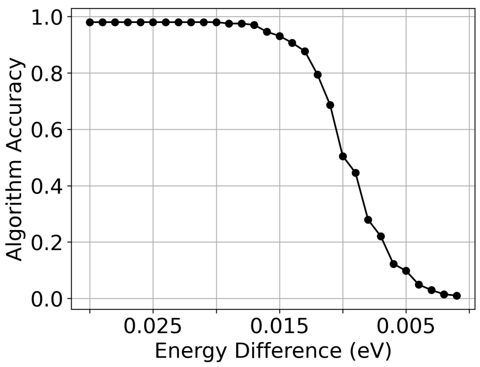

(b) Algorithm Accuracy

Fig. S-7: Band gap autocharacterization algorithm performance. (a) Direct band gap values output by the automatic band gap extraction algorithm, split by batch, as a function of formamidinium (FA) and methylammonium (MA) mixed-cation $FA_{1-x}MA_xPbI_3$ composition. (b) Automatic band gap extraction algorithm accuracy as a function of the maximum allowable difference in energy between the domain expert-calculated and automatically calculated band gaps.

Figure S-7a illustrates the autocharacterization output band gaps as a function of composition for the three independent batches. Figure S-7b illustrates the accuracy of the automatic algorithm as a function of the energy difference threshold. The algorithm achieves 98.5% accuracy within 0.02 eV and as the threshold becomes tighter, the algorithm accuracy is expected to decrease.

Stability

To conduct the degradation experiments in this paper, we put the samples in a degradation chamber and monitor the conditions for 2 hours, capturing RGB images (Thorlabs DCC1645C camera with the infrared filter removed to increase sensitivity towards dark samples) every 30 seconds. The construction and operation of the degradation chamber setup are detailed in Keesey & Tiihonen *et al.* [6]. Figure S-8(a) shows the time series of temperature and humidity conditions over the course of the experiment. Over the 2-hour degradation experiment, the temperature conditions were maintained at 34.5°C ± 0.5°C with a relative humidity of 40% ± 1%. An initial jump in temperature with a respective dip in humidity is noted as the samples are placed into the degradation chamber before the internal environment equilibrates. A class AAA solar simulator (G2V Optics Sunbrick Base, with visible-only part of the AM1.5G spectrum) is used to illuminate the 3"×2" substrate of samples during the

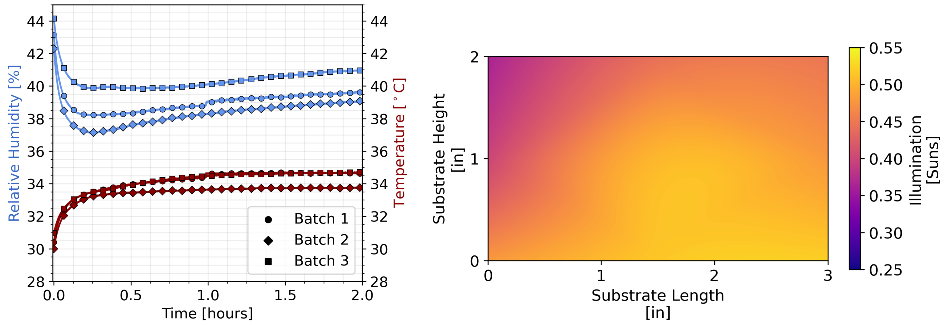

(a) Temporal Degradation Conditions (b) Fixed Degradation Illumination

Fig. S-8: Perovskite degradation environmental conditions. (a) Temporal degradation conditions for each of the 3 batches of samples over the course of 2 hours, measured using a temperature and humidity sensor (Adafruit, AHT10). (b) Spatial uniformity of illumination measured across the substrate within the degradation chamber, measured using a lux sensor (Adafruit, VEML7700).

2-hour degradation experiment. Figure S-8(b) shows the spatial uniformity of the illumination across the substrate internal to the degradation chamber, measured using a lux sensor (Adafruit, VEML7700). These illumination conditions are held fixed over the course of the experiment. Most of the substrate experiences 0.50-0.55 suns of illumination, however, in the top-left corner of the substrate, a dip in illumination occurs as a result of minor occlusion due to sensor placement. Figure S-9 illustrates that this dip in illumination at the top-left corner of the substrate does not have a major effect on the degradation pattern of the $FA_{1-x}MA_xPbI_3$ compositional series. Degradation, as indicated by the yellowing, is shown to begin in the formamidinium (FA)-rich end of the samples and migrates towards the methylammonium (MA)-rich end over time.

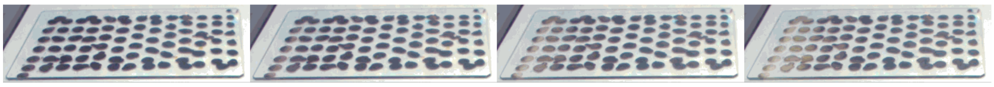

Fig. S-9: Degradation of the high-throughput manufactured formamidinium (FA) and methylammonium (MA) mixed-cation gradient $FA_{1-x}MA_xPbI_3$ over 2 hours.

Inhomogeneities in the degradation process can be accounted for when computing the degradation intensity, I_c. Figure S-10 illustrates the I_c over time but computed for every pixel across three different samples: (a) a fully degraded sample, (b) a sample with spotted degradation, and (c) a sample that did not degrade. The red dashed line shows the spatial average for that sample, which, at the final timestep, is the actual I_c value output by the algorithm. For the non-degraded sample, the distribution of I_c values across pixels is tight since the color values change very little over the experiment. Similarly, the fully degraded sample has a mostly tight distribution since the entire

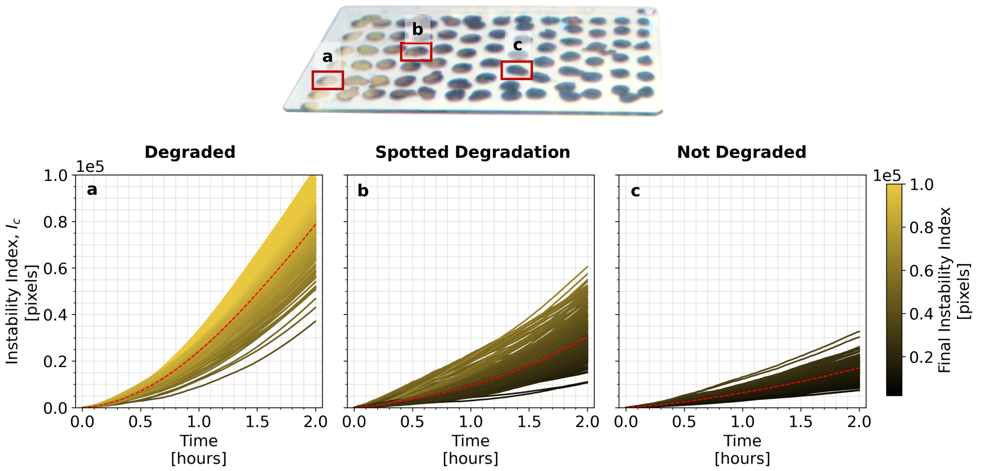

Fig. S-10: Instability index computed for each pixel for three different perovskite material samples. (a) Fully degraded perovskite, (b) spotted degradation perovskite, (c) not degraded perovskite. Each line plot indicates the change in the instability index over the course of the degradation experiment. The colormap visualizes the final instability index at the end of the experiment for that pixel; yellow indicates high degradation and black indicates low degradation. The red dashed line shows the spatial average of instability index across all pixels for that material, corresponding to the true instability index computed by the autocharacterization algorithm.

sample degrades uniformly. Conversely, for the sample that experiences a spotted and inhomogeneous degradation pattern, the spread of I_c values since both non-degraded (low I_c) and highly degraded (high I_c) pixels are included. This inhomogeneous degradation may arise from thickness variations, coffee ring effects, or other defects [7, 8]. Thus, due to high I_c values representing regions of degradation in the spatial average, it is shown that even for samples experiencing spotted and inhomogenous degradation, the overall intensity pattern is captured.

After segmenting each sample across the entire time domain of the experiment, the full matrix of degradation time series is populated and color calibrated automatically, as shown in Figure S-11. In this specific experiment, spatial non-uniformity of reflected surfaces was detected in the post-analysis, which does not noticeably affect the instability index calculation but does give rise to artificial color differences in the samples, depending on their location on the substrate. These spatially-dependent color differences arise due to the physical configuration of the environmental chamber and the RGB camera. To account for these color differences in the final output matrix time series, an additional color correction step was applied that initializes all deposited samples to the same color. This color correction is only cosmetic and was not used in the calculations for determining the degradation intensity, I_c. Color correction aids in the interpretation of the visualized time series data by making the color changes due to degradation easier to see while diminishing the unwanted effects of spatially-dependent reflectivity aberrations. After the color correction, a fully calibrated matrix

of degradation time-series is acquired, showing the color change over time for each perovskite sample within its batch, shown in Figure S-11.

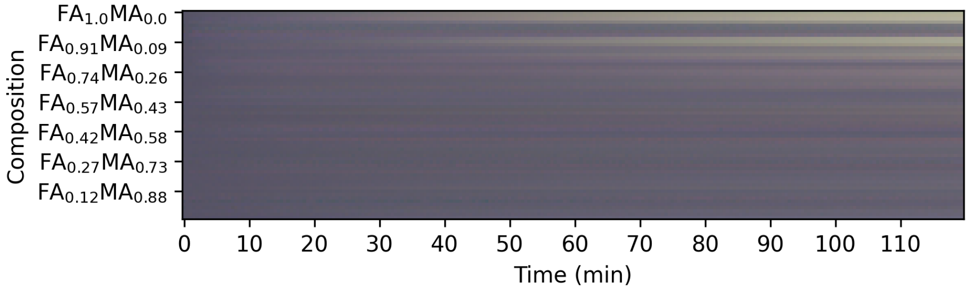

(a) Stability Time Series for Batch 1

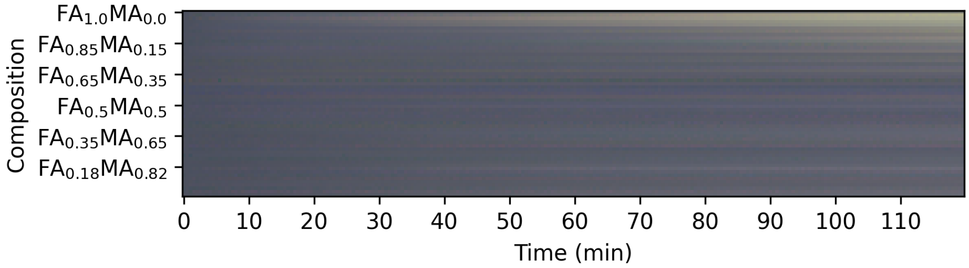

(b) Stability Time Series for Batch 2

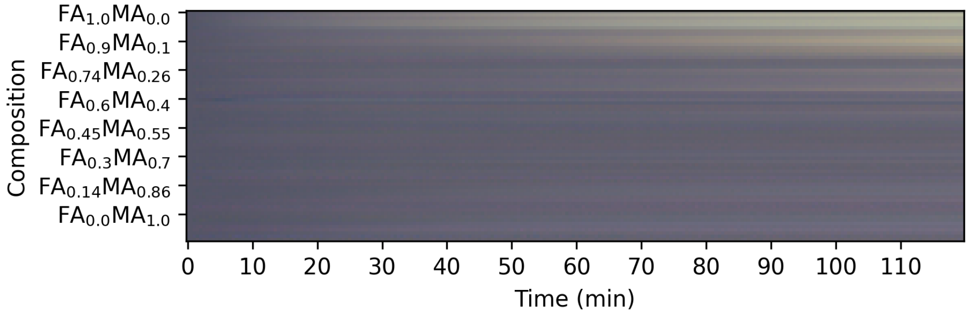

(c) Stability Time Series for Batch 3

Fig. S-11: Final stability time series matrix of all batches of segmented droplets over the course of a 2-hour degradation experiment. All samples begin the experiment colored as dark gray and over the course of degradation, samples with methylammonium (MA) proportions between 0% to 20% exhibit yellowing around the 20 min to 40 min mark, thus indicating degradation. Figure panels represent the temporal degradation of uniquely synthesized batches: (a) batch 1, (b) batch 2, and (c) batch 3.

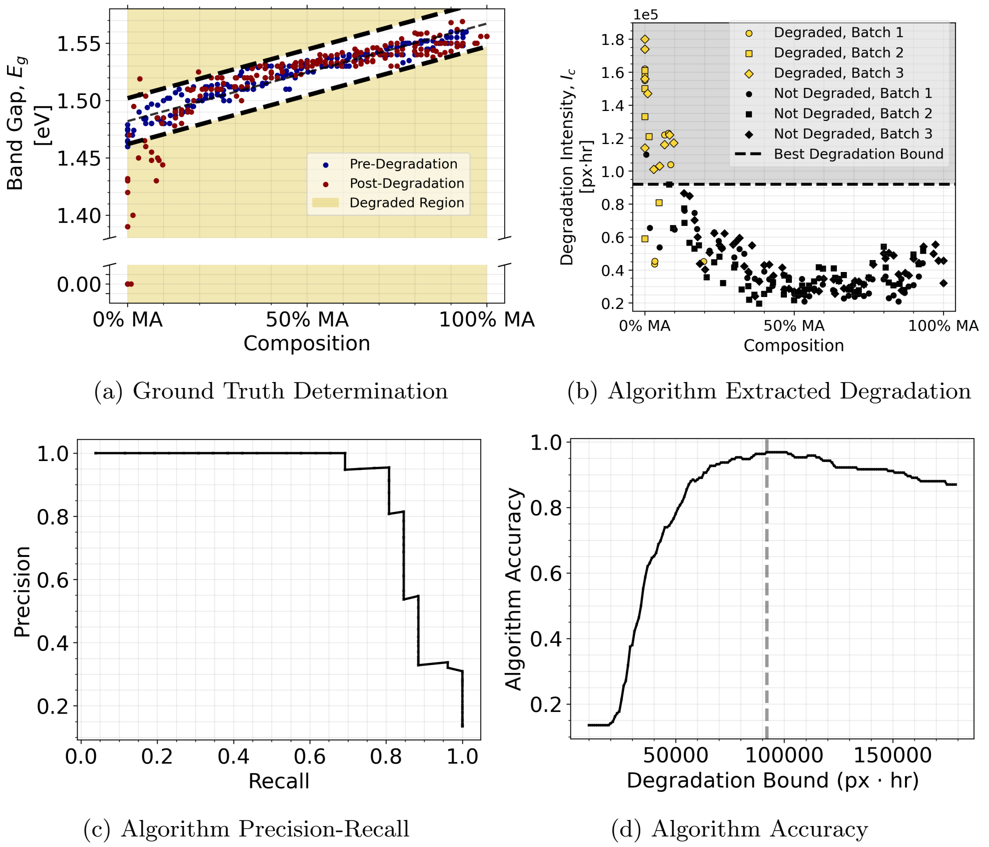

(a) Ground Truth Determination

(b) Algorithm Extracted Degradation

(c) Algorithm Precision-Recall

(d) Algorithm Accuracy

Fig. S-12: Automatic degradation detection algorithm performance benchmarking. (a) Shows how the ground truth degradation is determined using post-degradation band gap measurement. The break in the graph is used to visualize which compositions had no band gap after degradation, reported as 0.0 eV. (b) Values of instability index, I_c, as a function of composition, split by batch with a total of $N = 201$ samples. I_c is used as a classifier for degradation, where the dashed line indicates the separation of high I_c versus low I_c. (c) Precision-recall performance of I_c as a classifier for degradation based on the classification rate of false negatives and false positives. (d) Accuracy of I_c as a classifier for determining high and low degradation. The x-axis indicates the decision boundary value of I_c, where values above are considered degraded and values below are not. The optimal value of I_c as a decision boundary for degradation is $I_c = 0.92 \times 10^5$ px·hr, and is shown as a dashed vertical line. This value is where the accuracy of the algorithm is maximum at 96.9%.

The degradation intensity, I_c, is computed from the color-changing time series per sample using Equation 3 [6, 9]. The degradation detection algorithm automatically computes I_c for every sample. High values of I_c correspond to high degradation. To

benchmark the algorithm, the difference in domain expert-computed band gaps before and after degradation is used as a ground truth to quantify whether the material has truly degraded or not. This is possible due to the change in band gap that occurs in perovskites during either a phase transition or chemical decomposition [9, 10]. Hence, in Figure S-12(a), any expert-calculated post-degraded band gaps (red points) samples that fall outside of the ±0.02 eV bounds, with respect to the regression fit line to the expert-calculated pre-degradation band gaps (blue points), are considered to exhibit “Ground Truth Degradation”. These ground truth-degraded samples are denoted by yellow scatter points in Figure S-12(b). The bounds of ±0.02 eV are used because they empirically fit the expert-calculated pre-degradation band gaps (blue points) with little to no tolerance. Thus, the region where “Ground Truth Degradation” occurs in the post-degraded samples is indicated by the yellow shaded region in Figure S-12(a).

Figure S-12(b) illustrates that the magnitude of I_c strongly corresponds with the ground truth determination of degradation using band gap difference as a metric. This correspondence can be quantified using the precision-recall (PR) of the autocharacterization algorithm. A PR curve quantifies the performance of using a classifier, in this case, I_c, to predict a ground truth, in this case, degradation:

$$\begin{aligned} \text{Recall} &= \frac{\text{TP}}{\text{TP} + \text{FN}} \\ \text{Precision} &= \frac{\text{TP}}{\text{TP} + \text{FP}}, \end{aligned} \tag{S-5}$$

where TP are the true positives, FN are the false negatives, FP are the false positives. We use the PR curve instead of the ROC (receiver operating characteristic) curve here due to the large class imbalance between the number of degraded samples versus non-degraded samples (there are significantly more non-degraded samples than there are degraded samples).

Figure S-12c illustrates the PR curve of the automatic degradation detection algorithm based on the degradation decision boundary (horizontal black dashed line in Figure S-12b). The goal is to have both high precision and high recall simultaneously. The PR-AUC (precision-recall area under the curve) figure of merit boils the PR curve down to a single number that determines the performance of I_c as a good predictor for degradation. The value of PR-AUC falls between 0.0 and 1.0, where a value of 1.0 represents perfect performance. The I_c values computed by the autocharacterization algorithm achieve a PR-AUC of $0.853 \in [0, 1]$, implying that high values of I_c do strongly correspond to ground truth degradation. Figure S-12d shows the effect of moving the decision boundary on the accuracy in detecting the degradation. Considering recall, precision, and accuracy, I_c performs optimally, with an accuracy of 96.9%, in detecting degraded samples when the decision boundary is set to 0.92×10^5 px·hr. However, accuracies of over 90% are achieved for a wide range of I_c decision boundaries: 0.7×10^5 px·hr $\leq I_c \leq 1.6 \times 10^5$ px·hr. Hence, indicating that I_c is a general yet strong predictor of degradation.

Phase and Elemental Analysis

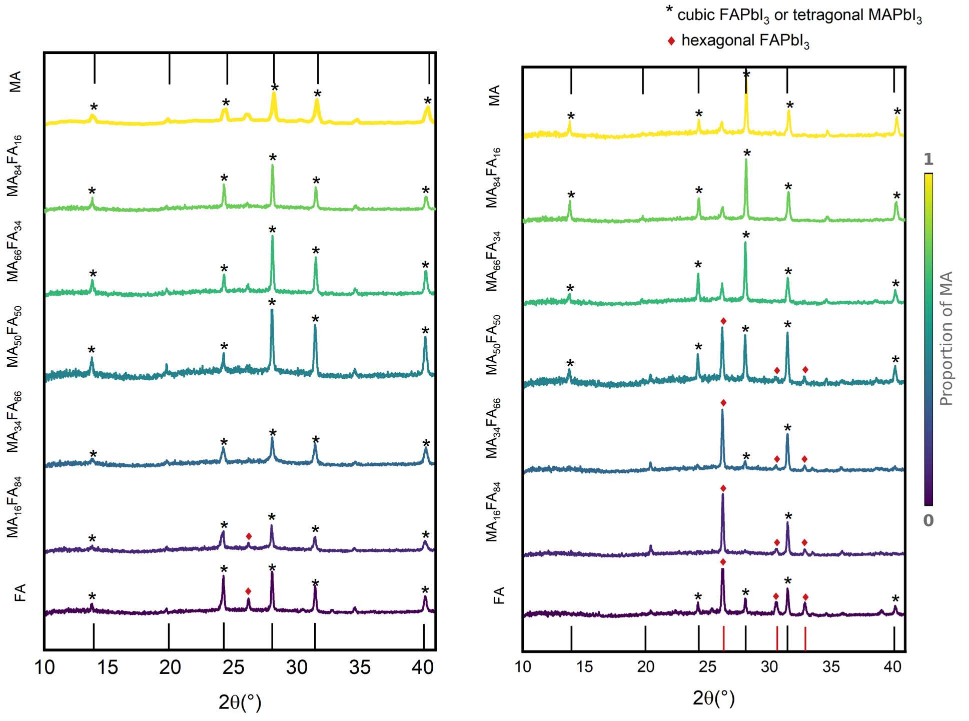

(a) Pre-degradation XRD

(b) Post-degradation XRD

Fig. S-13: XRD peak intensities for uniformly-spaced compositions along the formamidinium (FA) and methylammonium (MA) mixed-cation perovskite system $FA_{1-x}MA_xPbI_3$ (a) before and (b) after degradation.

Phase analysis, such as X-ray diffraction (XRD), of perovskites is used to determine the structure and quality of the manufactured samples. In this study, we measure our samples using the Bruker X-ray Diffractometer with a Cobalt Source D8 and General Area Detector Diffraction System. Figure S-13 illustrates the pre- and post-degradation XRD traces for equally spaced compositions along the $FA_{1-x}MA_xPbI_3$ series. The reference peak locations for both the favorable cubic α-$FAPbI_3$ [11] and favorable tetragonal $MAPbI_3$ phases [12] are shown together as black vertical lines and "$*$" symbols since a shift of only $\Delta 2\theta \approx 0.16°$ is seen from $FAPbI_3$ to $MAPbI_3$ at around the $2\theta = 31.5°$ peak. High-resolution scans of this peak shift along $FA_{1-x}MA_xPbI_3$ are shown in Figure 3b and can be used as an additional validation tool for composition shift. During degradation, $FAPbI_3$ phase transitions from a favorable cubic α-phase to a non-perovskite hexagonal δ-phase [11]. Hence, XRD is a useful validation tool for pre- and post-degradation determination. Prior to degradation, Figure S-13a illustrates that the samples exhibit high adherence to their favorable

phases with only minor contribution of δ-$FAPbI_3$ phase. However, after the degradation test, Figure S-13b illustrates high degradation (indicated by the high peak intensities of the δ-$FAPbI_3$ phase at $2\theta = 26.3°$, denoted by the red diamond) in the FA-rich compositions. This XRD result matches the detected yellowing degradation pattern of the FA-rich samples, as shown in Figure S-9 and Figure S-11.

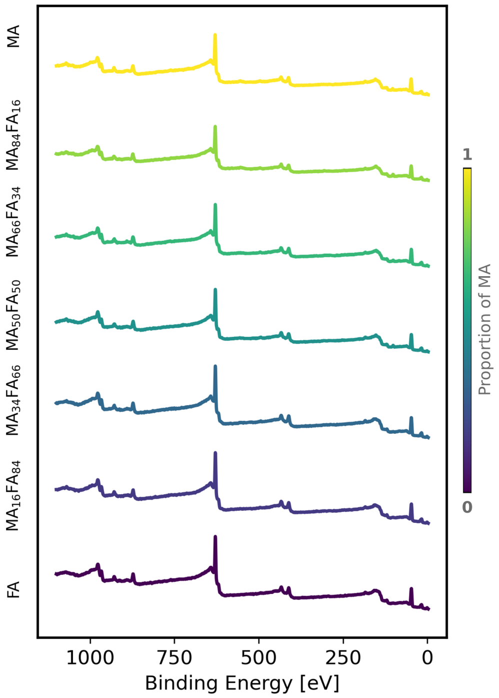

Fig. S-14: Full XPS spectra for uniformly-spaced compositions along the formamidinium (FA) and methylammonium (MA) mixed-cation perovskite system $FA_{1-x}MA_xPbI_3$.

Elemental analysis, such as X-ray photoelectron spectroscopy (XPS), is used to determine the shift in the binding energy of bonds present within the different A-site cations (FA and MA) along the $FA_{1-x}MA_xPbI_3$ series, in turn corresponding to a "composition" shift. XPS is a surface-sensitive quantitative spectroscopic technique that can identify crystalline phases. In this study, we measure our samples using the PHI 5000 Versa Probe II Focus X-ray Photoelectron Spectrometer, equipped with a monochromated AlKα X-ray source for excitation at 1486.6 eV with an X-ray beam size of 200 μm. Survey spectra of $FAPbI_3$ to $MAPbI_3$ series are depicted in Figure S-14. Using the survey spectra alone, there are variations in A-site composition that can not be distinguished clearly in survey spectra due to the many similarities between the FA and MA molecules [13]. However, the primary distinguishing feature of these is the presence of the carbon-nitrogen double bond (C=N), clearly detectable in the

high-resolution XPS scans, as shown in Figure 3c. In the high-resolution scans of *C1s1* and *N1s2*, the calibration is performed on the lowest *C1s* energy peak of 284.8 eV. Hence, the shift in C=N peak intensity quantifies the presence of FA relative to MA, in turn, determining composition along the $FA_{1-x}MA_xPbI_3$ series.

Figure S-15 shows the quantitative XRD peak shifts and XPS peak intensities from the high-resolution scans for the phase and elemental shifts that occur along the $FA_{1-x}MA_xPbI_3$ series. The XRD peak of the (012) crystallographic plane shifts from lower to higher 2θ angles as more MA is added to the composition. Conversely, the XPS peak for the presence of C=N bonds shifts from higher to lower intensity as more MA is added to the composition. Thus, both of these measurements validate the presence of a compositional gradient occurring across the synthesized batches of samples.

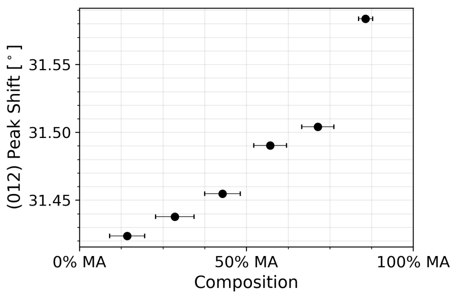

(a) XRD (012) Peak Angle Shift

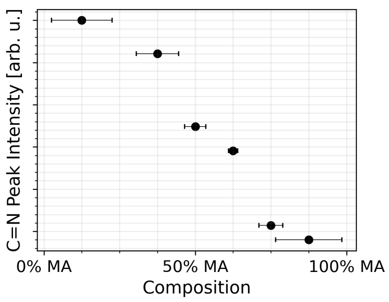

(b) XPS C=N Peak Intensity Shift

Fig. S-15: XRD and XPS peaks for each measured composition. (a) XRD 2θ values for the (012) crystallographic plane peak shift from FA-rich to MA-rich formamidinium (FA) and methylammonium (MA) mixed-cation compositions $FA_{1-x}MA_xPbI_3$. (b) XPS intensity shift for the C=N peak from FA-rich to MA-rich $FA_{1-x}MA_xPbI_3$ compositions. The horizontal error bars illustrate the relative widths of the XRD and XPS peaks.

Size of the Perovskite Search Space

A commonly explored metal halide perovskite search space for photovoltaic applications from literature consists of the following eight-component material system: $(FA_xMA_yCs_{1-x-y})(Pb_zSn_{1-z})(Br_aCl_bI_{1-a-b})_3$ [9, 14–18]. Figure S-16 shows the discretization of these eight components within the archetypal ABX_3 perovskite structure. The number of steps per edge, n, determines the compositional resolution for each subspace. As the number of steps increases, the number of potential compositions increases, and, in turn, the search space becomes more vast. For this eight-component search space, the number of possible compositions is proportional to the product of each subspace's (A, B, and X) step size to the power of the number of components

within each subspace (here, 3-components for A (FA, MA, and Cs), 2-components for B (Pb and Sn), and 3-components for X (Br, Cl, and I)) $\implies$ $n^3 \times n \times n^3$ for the A×B×X subspaces. A caveat in this equation is that for binary subspaces, the power of the step size is 1 instead of 2 since the space is linear, as shown by the B-site subspace in Figure S-16. Hence, for a low-resolution search space of $n = 10$ steps, 1×10^6 total compositions are considered; and for a high-resolution search space of $n = 100$ steps, 7×10^{12} compositions are considered.

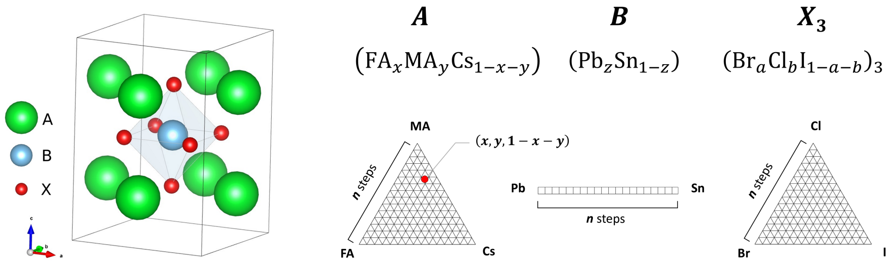

Fig. S-16: Archetypal metal halide perovskite compositional search space.

Full Experimental Results

Table S-1 contains the full readout of the characterization results extracted by the autocharacterization algorithms for all $N = 201$ samples. We report the numerical values of calculated composition, autocharacterization-calculated band gap (Auto E_g), human domain expert-calculated band gap (Expert E_g), autocharacterization-calculated degree of degradation (I_c), and the ground truth degradation determined by the human domain expert. Dashes indicate values unable to be determined for that composition due to missing data.

Table S-1: Full readout of characterization results for all 201 perovskite materials synthesized in this study. Columns of the results include: unique sample number, composition of the material in the format $FA_{1-x}MA_xPbI_3$ (formamidinium: FA, methylammonium: MA, lead: Pb, iodine: I), algorithm-computed band gap in electron volts (eV), expert-measured band gap in electron volts (eV), algorithm-computed instability index (Ic) in pixel hours (px-hr), and the ground truth determination of degradation, determined by a domain expert. Missing data values are shown as a dash. This table is provided as a Supplementary Data file with this paper.

Sample	Computed Composition	Auto E_g (eV)	Expert E_g (eV)	I_c (px·hr)×10^5	Degradation (Ground Truth)
1	$FA_{1.000}MA_{0.000}PbI_3$	1.471	1.460	1.581	Yes
2	$FA_{1.000}MA_{0.000}PbI_3$	1.463	1.474	1.737	Yes

Table S-1: Continued from previous page.

Sample	Computed Composition	Auto E_g (eV)	Expert E_g (eV)	I_c (px·hr)$\times10^5$	Degradation (Ground Truth)
3	$FA_{1.000}MA_{0.000}PbI_3$	1.463	1.475	1.562	Yes
4	$FA_{1.000}MA_{0.000}PbI_3$	1.467	1.479	1.799	Yes
5	$FA_{1.000}MA_{0.000}PbI_3$	1.458	1.464	1.335	Yes
6	$FA_{1.000}MA_{0.000}PbI_3$	1.469	1.470	1.739	Yes
7	$FA_{1.000}MA_{0.000}PbI_3$	1.454	1.460	1.465	Yes
8	$FA_{1.000}MA_{0.000}PbI_3$	1.455	1.470	-	Yes
9	$FA_{1.000}MA_{0.000}PbI_3$	1.450	1.460	-	Yes
10	$FA_{1.000}MA_{0.000}PbI_3$	1.457	1.470	-	Yes
11	$FA_{1.000}MA_{0.000}PbI_3$	1.457	1.465	1.607	Yes
12	$FA_{1.000}MA_{0.000}PbI_3$	1.475	1.475	1.501	No
13	$FA_{1.000}MA_{0.000}PbI_3$	1.473	1.470	1.098	Yes
14	$FA_{1.000}MA_{0.000}PbI_3$	1.471	1.470	1.623	Yes
15	$FA_{0.987}MA_{0.013}PbI_3$	1.464	1.470	1.209	Yes
16	$FA_{0.983}MA_{0.017}PbI_3$	1.460	1.474	0.655	No
17	$FA_{0.968}MA_{0.032}PbI_3$	1.488	1.490	0.436	Yes
18	$FA_{0.955}MA_{0.045}PbI_3$	1.479	1.484	0.453	Yes
19	$FA_{0.950}MA_{0.050}PbI_3$	1.474	1.480	0.809	Yes
20	$FA_{0.950}MA_{0.050}PbI_3$	1.475	1.484	0.538	No
21	$FA_{0.950}MA_{0.050}PbI_3$	1.478	1.485	1.012	Yes
22	$FA_{0.945}MA_{0.055}PbI_3$	1.490	1.494	1.034	Yes
23	$FA_{0.933}MA_{0.067}PbI_3$	1.471	1.480	1.215	Yes
24	$FA_{0.933}MA_{0.067}PbI_3$	1.470	1.480	1.165	Yes
25	$FA_{0.933}MA_{0.067}PbI_3$	1.494	1.497	1.223	Yes
26	$FA_{0.917}MA_{0.083}PbI_3$	1.473	1.484	1.040	Yes
27	$FA_{0.917}MA_{0.083}PbI_3$	1.474	1.485	0.918	No
28	$FA_{0.917}MA_{0.083}PbI_3$	1.492	1.496	1.231	Yes
29	$FA_{0.907}MA_{0.093}PbI_3$	1.473	1.482	0.652	No
30	$FA_{0.900}MA_{0.100}PbI_3$	1.464	1.480	1.170	Yes
31	$FA_{0.900}MA_{0.100}PbI_3$	1.466	1.485	-	-
32	$FA_{0.900}MA_{0.100}PbI_3$	1.494	1.500	0.645	No
33	$FA_{0.898}MA_{0.102}PbI_3$	1.471	1.480	-	-
34	$FA_{0.889}MA_{0.111}PbI_3$	1.494	1.502	-	-
35	$FA_{0.883}MA_{0.117}PbI_3$	1.470	1.480	-	-
36	$FA_{0.883}MA_{0.117}PbI_3$	1.494	1.502	-	-
37	$FA_{0.881}MA_{0.119}PbI_3$	1.478	1.487	-	No
38	$FA_{0.867}MA_{0.133}PbI_3$	1.496	1.507	0.759	No
39	$FA_{0.867}MA_{0.133}PbI_3$	1.474	1.490	0.773	No
40	$FA_{0.867}MA_{0.133}PbI_3$	1.475	1.487	0.684	No
41	$FA_{0.857}MA_{0.143}PbI_3$	1.502	1.510	0.866	-
42	$FA_{0.850}MA_{0.150}PbI_3$	1.494	1.505	0.851	No
43	$FA_{0.850}MA_{0.150}PbI_3$	1.477	1.490	0.565	No

Table S-1: Continued from previous page.

Sample	Computed Composition	Auto E_g (eV)	Expert E_g (eV)	I_c (px·hr)×10^5	Degradation (Ground Truth)
44	$FA_{0.850}MA_{0.150}PbI_3$	1.482	1.490	0.849	No
45	$FA_{0.846}MA_{0.154}PbI_3$	1.481	1.490	0.438	No
46	$FA_{0.833}MA_{0.167}PbI_3$	1.487	1.495	0.529	No
47	$FA_{0.833}MA_{0.167}PbI_3$	1.493	1.500	0.703	No
48	$FA_{0.833}MA_{0.167}PbI_3$	1.503	1.510	0.747	No
49	$FA_{0.819}MA_{0.181}PbI_3$	1.484	1.495	0.649	No
50	$FA_{0.817}MA_{0.183}PbI_3$	1.503	1.512	0.549	No
51	$FA_{0.817}MA_{0.183}PbI_3$	1.490	1.495	0.600	No
52	$FA_{0.803}MA_{0.197}PbI_3$	1.494	1.500	0.452	Yes
53	$FA_{0.792}MA_{0.208}PbI_3$	1.491	1.500	0.354	No
54	$FA_{0.783}MA_{0.217}PbI_3$	1.492	1.502	0.403	No
55	$FA_{0.783}MA_{0.217}PbI_3$	1.502	1.512	0.518	No
56	$FA_{0.782}MA_{0.218}PbI_3$	1.498	1.505	0.504	No
57	$FA_{0.779}MA_{0.221}PbI_3$	1.503	1.512	-	-
58	$FA_{0.767}MA_{0.233}PbI_3$	1.494	1.503	0.446	No
59	$FA_{0.767}MA_{0.233}PbI_3$	1.508	1.513	0.628	No
60	$FA_{0.767}MA_{0.233}PbI_3$	1.494	1.505	0.623	No
61	$FA_{0.750}MA_{0.250}PbI_3$	1.494	1.508	0.480	No
62	$FA_{0.750}MA_{0.250}PbI_3$	1.506	1.514	0.576	No
63	$FA_{0.750}MA_{0.250}PbI_3$	1.492	1.505	0.551	No
64	$FA_{0.738}MA_{0.262}PbI_3$	1.493	1.504	0.532	No
65	$FA_{0.733}MA_{0.267}PbI_3$	1.487	1.505	0.531	No
66	$FA_{0.733}MA_{0.267}PbI_3$	1.503	1.514	0.320	No
67	$FA_{0.733}MA_{0.267}PbI_3$	1.497	1.509	0.621	No
68	$FA_{0.733}MA_{0.267}PbI_3$	1.490	1.500	-	-
69	$FA_{0.724}MA_{0.276}PbI_3$	1.504	1.514	-	-
70	$FA_{0.717}MA_{0.283}PbI_3$	1.495	1.500	-	-
71	$FA_{0.717}MA_{0.283}PbI_3$	1.503	1.512	-	-
72	$FA_{0.712}MA_{0.288}PbI_3$	1.498	1.512	0.555	No
73	$FA_{0.700}MA_{0.300}PbI_3$	1.505	1.512	0.477	No
74	$FA_{0.700}MA_{0.300}PbI_3$	1.499	1.510	0.414	No
75	$FA_{0.697}MA_{0.303}PbI_3$	1.501	1.510	0.433	No
76	$FA_{0.686}MA_{0.314}PbI_3$	1.511	1.522	0.567	No
77	$FA_{0.683}MA_{0.317}PbI_3$	1.502	1.510	0.595	-
78	$FA_{0.683}MA_{0.317}PbI_3$	1.503	1.510	0.528	No
79	$FA_{0.683}MA_{0.317}PbI_3$	1.514	1.525	-	No
80	$FA_{0.677}MA_{0.323}PbI_3$	1.502	1.510	0.282	No
81	$FA_{0.667}MA_{0.333}PbI_3$	1.516	1.528	0.498	No
82	$FA_{0.667}MA_{0.333}PbI_3$	1.504	1.515	0.423	No
83	$FA_{0.667}MA_{0.333}PbI_3$	1.498	1.510	0.219	No
84	$FA_{0.650}MA_{0.350}PbI_3$	1.501	1.510	0.353	No

Table S-1: Continued from previous page.

Sample	Computed Composition	Auto E_g (eV)	Expert E_g (eV)	I_c (px·hr)×10^5	Degradation (Ground Truth)
85	$FA_{0.650}MA_{0.350}PbI_3$	1.502	1.510	0.383	No
86	$FA_{0.650}MA_{0.350}PbI_3$	1.507	1.515	0.302	No
87	$FA_{0.625}MA_{0.375}PbI_3$	1.503	1.513	0.250	No
88	$FA_{0.624}MA_{0.376}PbI_3$	1.510	1.515	0.389	No
89	$FA_{0.617}MA_{0.383}PbI_3$	1.509	1.520	0.335	No
90	$FA_{0.614}MA_{0.386}PbI_3$	1.505	1.515	0.196	No
91	$FA_{0.611}MA_{0.389}PbI_3$	1.522	1.530	0.549	No
92	$FA_{0.600}MA_{0.400}PbI_3$	1.518	1.530	0.391	No
93	$FA_{0.600}MA_{0.400}PbI_3$	1.509	1.520	0.255	No
94	$FA_{0.600}MA_{0.400}PbI_3$	1.509	1.520	0.241	No
95	$FA_{0.583}MA_{0.417}PbI_3$	1.510	1.520	0.331	No
96	$FA_{0.583}MA_{0.417}PbI_3$	1.511	1.522	0.280	No
97	$FA_{0.583}MA_{0.417}PbI_3$	1.518	1.530	0.344	No
98	$FA_{0.569}MA_{0.431}PbI_3$	1.512	1.523	0.303	No
99	$FA_{0.567}MA_{0.433}PbI_3$	1.520	1.530	0.325	No
100	$FA_{0.567}MA_{0.433}PbI_3$	1.508	1.520	0.429	No
101	$FA_{0.559}MA_{0.441}PbI_3$	1.501	1.510	0.433	No
102	$FA_{0.558}MA_{0.442}PbI_3$	1.512	1.526	0.366	No
103	$FA_{0.558}MA_{0.442}PbI_3$	1.511	1.523	0.320	No
104	$FA_{0.550}MA_{0.450}PbI_3$	1.524	1.530	0.414	No
105	$FA_{0.550}MA_{0.450}PbI_3$	1.503	1.510	0.270	No
106	$FA_{0.545}MA_{0.455}PbI_3$	1.513	1.523	0.365	No
107	$FA_{0.533}MA_{0.467}PbI_3$	1.517	1.532	0.372	No
108	$FA_{0.533}MA_{0.467}PbI_3$	1.509	1.520	0.288	No
109	$FA_{0.529}MA_{0.471}PbI_3$	1.515	1.526	0.252	No
110	$FA_{0.517}MA_{0.483}PbI_3$	1.529	1.532	0.282	No
111	$FA_{0.517}MA_{0.483}PbI_3$	1.523	1.530	0.340	No
112	$FA_{0.517}MA_{0.483}PbI_3$	1.513	1.528	0.356	No
113	$FA_{0.511}MA_{0.489}PbI_3$	1.528	1.532	0.286	No
114	$FA_{0.504}MA_{0.496}PbI_3$	1.514	1.530	0.270	No
115	$FA_{0.500}MA_{0.500}PbI_3$	1.500	1.535	0.254	No
116	$FA_{0.500}MA_{0.500}PbI_3$	1.518	1.530	0.216	No
117	$FA_{0.498}MA_{0.502}PbI_3$	1.518	1.525	0.253	No
118	$FA_{0.483}MA_{0.517}PbI_3$	1.522	1.525	0.244	No
119	$FA_{0.459}MA_{0.541}PbI_3$	1.524	1.530	0.210	No
120	$FA_{0.450}MA_{0.550}PbI_3$	1.523	1.530	0.241	No
121	$FA_{0.447}MA_{0.553}PbI_3$	1.520	1.530	0.306	No
122	$FA_{0.445}MA_{0.555}PbI_3$	1.530	1.537	0.282	No
123	$FA_{0.433}MA_{0.567}PbI_3$	1.523	1.530	0.331	No
124	$FA_{0.433}MA_{0.567}PbI_3$	1.527	1.533	0.305	No
125	$FA_{0.433}MA_{0.567}PbI_3$	1.529	1.537	0.288	No

Table S-1: Continued from previous page.

Sample	Computed Composition	Auto E_g (eV)	Expert E_g (eV)	I_c (px·hr)×10^5	Degradation (Ground Truth)
126	$FA_{0.417}MA_{0.583}PbI_3$	1.524	1.533	0.416	No
127	$FA_{0.417}MA_{0.583}PbI_3$	1.525	1.530	0.262	No
128	$FA_{0.417}MA_{0.583}PbI_3$	1.528	1.537	0.351	No
129	$FA_{0.402}MA_{0.598}PbI_3$	1.524	1.533	0.312	No
130	$FA_{0.400}MA_{0.600}PbI_3$	1.518	1.530	0.248	No
131	$FA_{0.400}MA_{0.600}PbI_3$	1.521	1.530	0.287	No
132	$FA_{0.400}MA_{0.600}PbI_3$	1.527	1.539	0.408	No
133	$FA_{0.392}MA_{0.608}PbI_3$	1.524	1.530	0.279	No
134	$FA_{0.391}MA_{0.609}PbI_3$	1.527	1.535	0.271	No
135	$FA_{0.383}MA_{0.617}PbI_3$	1.531	1.538	0.347	No
136	$FA_{0.383}MA_{0.617}PbI_3$	1.517	1.530	0.457	No
137	$FA_{0.367}MA_{0.633}PbI_3$	1.529	1.539	0.274	No
138	$FA_{0.367}MA_{0.633}PbI_3$	1.527	1.535	0.410	No
139	$FA_{0.367}MA_{0.633}PbI_3$	1.530	1.540	0.278	No
140	$FA_{0.360}MA_{0.640}PbI_3$	1.529	1.540	0.352	No
141	$FA_{0.350}MA_{0.650}PbI_3$	1.529	1.540	0.332	No
142	$FA_{0.350}MA_{0.650}PbI_3$	1.528	1.535	0.256	No
143	$FA_{0.350}MA_{0.650}PbI_3$	1.531	1.540	0.347	No
144	$FA_{0.342}MA_{0.658}PbI_3$	1.531	1.540	0.335	No
145	$FA_{0.336}MA_{0.664}PbI_3$	1.529	1.535	0.291	No
146	$FA_{0.333}MA_{0.667}PbI_3$	1.532	1.542	0.227	No
147	$FA_{0.333}MA_{0.667}PbI_3$	1.532	1.542	0.369	No
148	$FA_{0.332}MA_{0.668}PbI_3$	1.529	1.540	0.289	No
149	$FA_{0.317}MA_{0.683}PbI_3$	1.532	1.540	0.244	No
150	$FA_{0.304}MA_{0.696}PbI_3$	1.535	1.542	0.283	No
151	$FA_{0.303}MA_{0.697}PbI_3$	1.531	1.542	0.281	No
152	$FA_{0.292}MA_{0.708}PbI_3$	1.530	1.540	0.236	No
153	$FA_{0.280}MA_{0.720}PbI_3$	1.533	1.546	0.254	No
154	$FA_{0.278}MA_{0.722}PbI_3$	1.537	1.545	0.266	No
155	$FA_{0.276}MA_{0.724}PbI_3$	1.529	1.540	0.276	No
156	$FA_{0.267}MA_{0.733}PbI_3$	1.538	1.548	0.243	No
157	$FA_{0.267}MA_{0.733}PbI_3$	1.532	1.548	0.312	No
158	$FA_{0.267}MA_{0.733}PbI_3$	1.530	1.540	0.305	No
159	$FA_{0.250}MA_{0.750}PbI_3$	1.537	1.547	0.258	No
160	$FA_{0.250}MA_{0.750}PbI_3$	1.534	1.540	0.333	No
161	$FA_{0.250}MA_{0.750}PbI_3$	1.538	1.547	0.354	No
162	$FA_{0.234}MA_{0.766}PbI_3$	1.537	1.548	0.344	No
163	$FA_{0.233}MA_{0.767}PbI_3$	1.530	1.540	0.331	No
164	$FA_{0.233}MA_{0.767}PbI_3$	1.538	1.550	0.441	No
165	$FA_{0.233}MA_{0.767}PbI_3$	1.525	1.533	0.312	No
166	$FA_{0.226}MA_{0.774}PbI_3$	1.538	1.550	0.353	No

Table S-1: Continued from previous page.

Sample	Computed Composition	Auto E_g (eV)	Expert E_g (eV)	I_c (px·hr)×10^5	Degradation (Ground Truth)
167	$FA_{0.221}MA_{0.779}PbI_3$	1.539	1.550	0.433	No
168	$FA_{0.217}MA_{0.783}PbI_3$	1.540	1.550	0.501	No
169	$FA_{0.217}MA_{0.783}PbI_3$	1.524	1.535	0.457	No
170	$FA_{0.200}MA_{0.800}PbI_3$	1.540	1.550	0.344	No
171	$FA_{0.200}MA_{0.800}PbI_3$	1.532	1.540	0.415	No
172	$FA_{0.200}MA_{0.800}PbI_3$	1.542	1.550	0.541	No
173	$FA_{0.191}MA_{0.809}PbI_3$	1.539	1.550	0.361	No
174	$FA_{0.183}MA_{0.817}PbI_3$	1.536	1.540	0.470	No
175	$FA_{0.183}MA_{0.817}PbI_3$	1.545	1.550	0.229	No
176	$FA_{0.183}MA_{0.817}PbI_3$	1.548	1.552	0.324	No
177	$FA_{0.172}MA_{0.828}PbI_3$	1.539	1.552	0.503	No
178	$FA_{0.167}MA_{0.833}PbI_3$	1.537	1.545	0.307	No
179	$FA_{0.167}MA_{0.833}PbI_3$	1.547	1.552	0.247	No
180	$FA_{0.166}MA_{0.834}PbI_3$	1.539	1.552	0.489	No
181	$FA_{0.163}MA_{0.837}PbI_3$	1.537	1.545	0.374	No
182	$FA_{0.150}MA_{0.850}PbI_3$	1.537	1.546	0.316	No
183	$FA_{0.150}MA_{0.850}PbI_3$	1.536	1.552	0.209	No
184	$FA_{0.133}MA_{0.867}PbI_3$	1.540	1.550	0.345	No
185	$FA_{0.125}MA_{0.875}PbI_3$	1.544	1.552	0.261	No
186	$FA_{0.117}MA_{0.883}PbI_3$	1.544	1.550	0.300	No
187	$FA_{0.112}MA_{0.888}PbI_3$	1.553	1.556	0.279	No
188	$FA_{0.111}MA_{0.889}PbI_3$	1.550	1.555	0.276	No
189	$FA_{0.109}MA_{0.891}PbI_3$	1.545	1.550	0.383	No
190	$FA_{0.100}MA_{0.900}PbI_3$	1.548	1.555	0.309	No
191	$FA_{0.100}MA_{0.900}PbI_3$	1.549	1.560	0.494	No
192	$FA_{0.100}MA_{0.900}PbI_3$	1.540	1.550	0.368	No
193	$FA_{0.083}MA_{0.917}PbI_3$	1.541	1.550	0.468	No
194	$FA_{0.083}MA_{0.917}PbI_3$	1.549	1.560	0.434	No
195	$FA_{0.083}MA_{0.917}PbI_3$	1.548	1.555	0.361	No
196	$FA_{0.067}MA_{0.933}PbI_3$	1.552	1.555	0.442	No
197	$FA_{0.067}MA_{0.933}PbI_3$	1.547	1.560	0.444	No
198	$FA_{0.067}MA_{0.933}PbI_3$	1.537	1.550	0.543	No
199	$FA_{0.067}MA_{0.933}PbI_3$	1.551	1.558	0.516	No
200	$FA_{0.067}MA_{0.933}PbI_3$	1.533	1.548	0.554	No
201	$FA_{0.058}MA_{0.942}PbI_3$	1.553	1.560	0.498	No

References

[1] Wang, T. *et al.* Indirect to direct bandgap transition in methylammonium lead halide perovskite. *Energy Environ. Sci.* **10**, 509–515 (2017).

[2] Targhi, F. F., Jalili, Y. S. & Kanjouri, F. Mapbi3 and fapbi3 perovskites as solar cells: Case study on structural, electrical and optical properties. *Results Phys.* **10**, 616–627 (2018).

[3] Kubelka, P. & Munk, F. A contribution to the optics of pigments. *Z. Technol. Phys.* **12**, 593–599 (1931).

[4] Makuła, P., Pacia, M. & Macyk, W. How to correctly determine the band gap energy of modified semiconductor photocatalysts based on uv-vis spectra. *J. Phys. Chem. Lett.* **9**, 6814–6817 (2018).

[5] Tauc, J., Grigorovici, R. & Vancu, A. Optical properties and electronic structure of amorphous germanium. *Phys. Status Solidi B* **15**, 627–637 (1966).

[6] Keesey, R. *et al.* An open-source environmental chamber for materials-stability testing using an optical proxy. *Digit. Discov.* (2023).

[7] Zhang, D., Li, D., Hu, Y., Mei, A. & Han, H. Degradation pathways in perovskite solar cells and how to meet international standards. *Commun. Mater.* **3**, 1–14 (2022).

[8] Zhang, Q. *et al.* Suppressing "coffee ring effect" to deposit high-quality cspbi3 perovskite films by drop casting. *Chem. Eng. J.* **454**, 140147 (2023).

[9] Sun, S. *et al.* A data fusion approach to optimize compositional stability of halide perovskites. *Matter* **4**, 1305–1322 (2021).

[10] Stoumpos, C. C., Mao, L., Malliakas, C. D. & Kanatzidis, M. G. Structure-band gap relationships in hexagonal polytypes and low-dimensional structures of hybrid tin iodide perovskites. *Inorg. Chem.* **56**, 56–73 (2017).

[11] Nan, Z. A. *et al.* Revealing phase evolution mechanism for stabilizing formamidinium-based lead halide perovskites by a key intermediate phase. *Chem* **7**, 2513–2526 (2021).

[12] Wu, J., Chen, J. & Wang, H. Phase transition kinetics of mapbi3 for tetragonal-to-orthorhombic evolution. *JACS Au* **3**, 1205–1212 (2023).

[13] Elsayed, M. R., Elseman, A. M., Abdelmageed, A. A., Hashem, H. M. & Hassen, A. Synthesis and numerical simulation of formamidinium-based perovskite solar cells: a predictable device performance at nis-egypt. *Sci. Reports* **13**, 1–16 (2023).

[14] Wang, T. *et al.* Sustainable materials acceleration platform reveals stable and efficient wide-bandgap metal halide perovskite alloys. *Matter* **6**, 2963–2986 (2023).

[15] Ahmadi, M., Ziatdinov, M., Zhou, Y., Lass, E. A. & Kalinin, S. V. Machine learning for high-throughput experimental exploration of metal halide perovskites. *Joule* **5**, 2797–2822 (2021).

[16] Wang, Z. *et al.* Efficient ambient-air-stable solar cells with 2d–3d heterostructured butylammonium-caesium- formamidinium lead halide perovskites. *Nat. Energy* **2**, 1–10 (2017).

[17] Sun, S. *et al.* Accelerated development of perovskite-inspired materials via high-throughput synthesis and machine-learning diagnosis. *Joule* **3**, 1437–1451 (2019).

[18] Liu, A. *et al.* High-performance metal halide perovskite transistors. *Nat. Electron.* 1–13 (2023).
