# Supplementary material for: Using scalable computer vision to automate high-throughput semiconductor characterization
Source: Nat Commun. 2024 Jun 11;15:4654. doi: 10.1038/s41467-024-48768-2 (PMC11166656; doi:10.1038/s41467-024-48768-2)
Supplement: Supplementary file 3 — Description of Additional Supplementary Files [file 41467_2024_48768_MOESM3_ESM.pdf]

File Name: Supplementary Data 1 (Table S-1)

Description: Full readout of characterization results for all 201 perovskite materials synthesized in this study. Columns of the results include: unique sample number, composition of the material in the format $FA_{1-x}MA_xPbI_3$ (formamidinium: FA, methylammonium: MA, lead: Pb, iodine: I), algorithm-computed band gap in electron volts (eV), expert-measured band gap in electron volts (eV), algorithm-computed instability index (I_c) in pixel hours (px-hr), and the ground truth determination of degradation, determined by the domain expert. Missing data values are shown as a dash.
